# Supplementary material for: Live-cell imaging uncovers the relationship between histone acetylation, transcription initiation, and nucleosome mobility
Source: Sci Adv. 2023 Oct 4;9(40):eadh4819. doi: 10.1126/sciadv.adh4819 (PMC10550241; doi:10.1126/sciadv.adh4819)
Supplement: Supplementary file 1 — Figs. S1 to S5 Legends for movies S1 to S3 [file sciadv.adh4819_sm.pdf]

Science Advances
AAAS

Supplementary Materials for

Live-cell imaging uncovers the relationship between histone acetylation, transcription initiation, and nucleosome mobility

Matthew N. Saxton *et al.*

Timothy J. Stasevich, tim.stasevich@colostate.edu

Sci. Adv. **9**, eadh4819 (2023)
DOI: 10.1126/sciadv.adh4819

The PDF file includes:

Figs. S1 to S5
Legends for movies S1 to S3

Other Supplementary Material for this manuscript includes the following:

Movies S1 to S3

Supplementary Figures

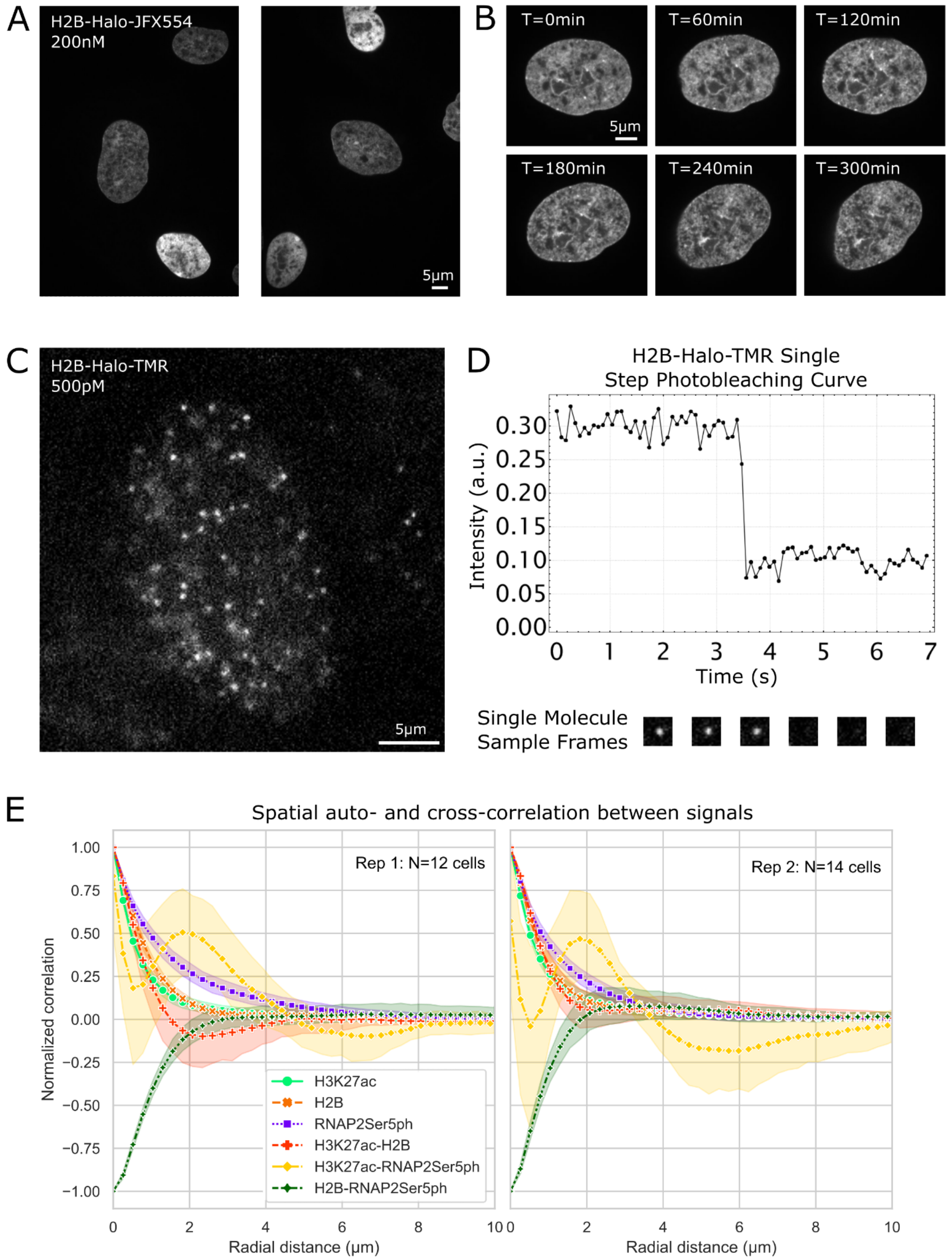

Fig. S1: H2B-Halo Stable RPE1 Cell Line Allows For Tracking Chromatin at Multiple Timescales

A. Sample images of multiple RPE1 cells in a single frame, stably expressing H2B-Halo, stained with Halo-JFX554 dye at 200 nM concentration.

B. Single cell H2B intensity and dynamics can be tracked for hours without substantial photobleaching

C. Same RPE1 cells as in A), except stained with 500 pM Halo-TMR. Now, single nucleosomes can be identified and tracked in real time with sub-second resolution.
D. Single-step photobleaching curve for a sample H2B track, along with frames of a tracked H2B before and after photobleach occurrence.
E. The spatial auto- and cross-correlation of the various signals. All were positively correlated except the cross-correlation between RNAP-Ser5ph and H2B. Note the significant peak in the cross-correlation between H3K27ac and RNAP2-Ser5ph, indicating the two signals are often offset from one another.

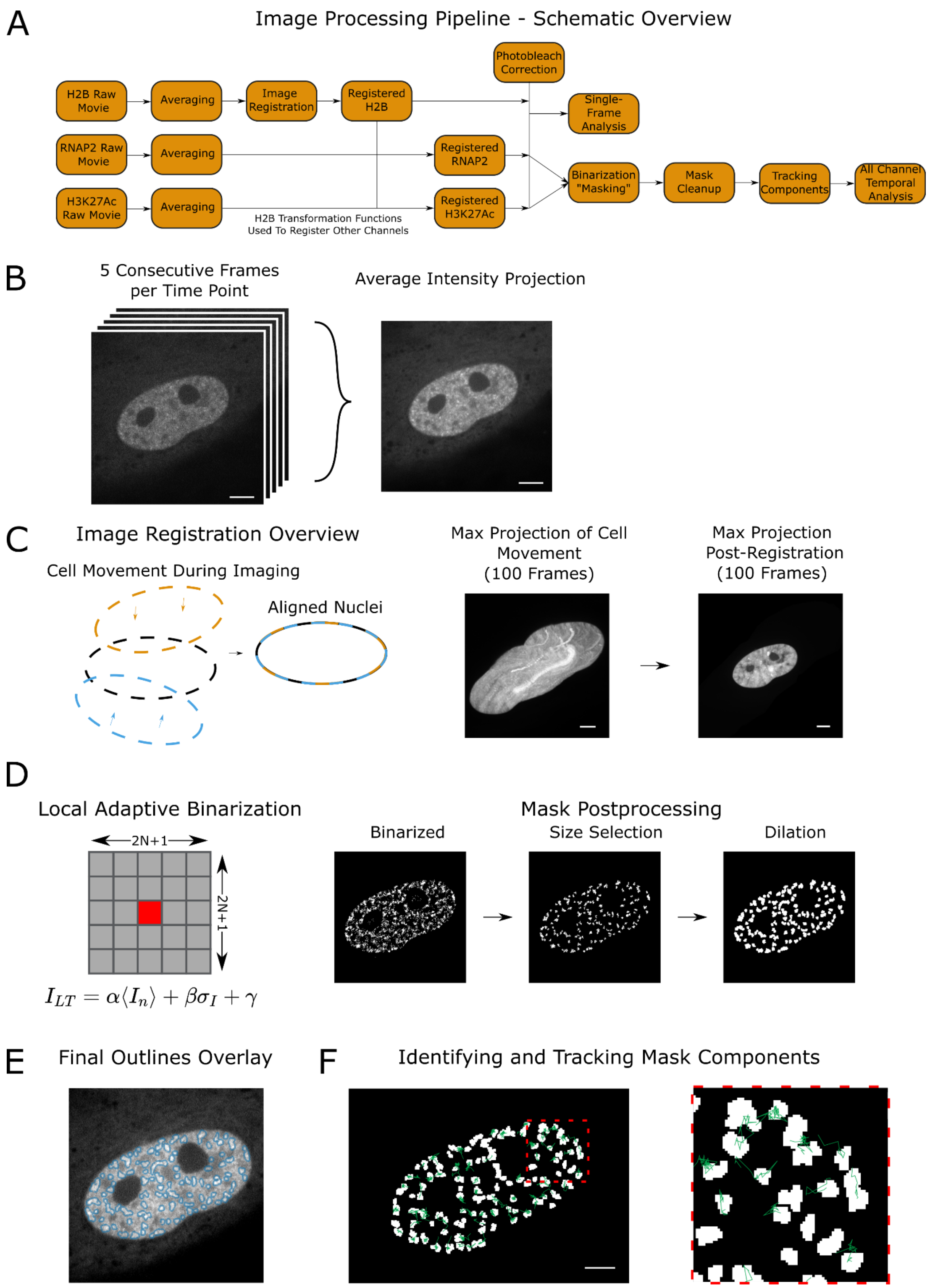

Fig. S2: Analysis pipeline for confocal imaging of RNAP2-Ser5ph, H2B, and H3K27ac in living RPE1 cells

A. Schematic overview of the analysis pipeline used to process confocal images and track regions of H3K27ac and RNAP2-Ser5ph.
B. Five frames are collected per time point and averaged to reduce noise from freely diffusing background Fab.

C. Each cell’s nucleus is individually registered to the H2B channel to correct for any cell movement during imaging. Pre- and Post-registration max projections of an example cell are shown.
D. Local adaptive binarization is used to identify regions of a nucleus enriched for H3K27ac or RNAP2-Ser5ph. For additional information see materials and methods. The same binarization parameters are applied to all cells in all cases. After binarization, components less than 10 pixels are removed to reduce speckle noise, and then dilated by a single pixel to fill in gaps and smooth edges.
E. An outline of final binarization components (blue) overlaid on the original Fab image.
F. Demonstration of tracking individual H3K27ac chromatin regions through time. Components with a line trace (green) show the detected region’s centroid movement over time. Components without a shown line trace did not meet the minimum requirement of 10 frame length for tracking and were discarded.

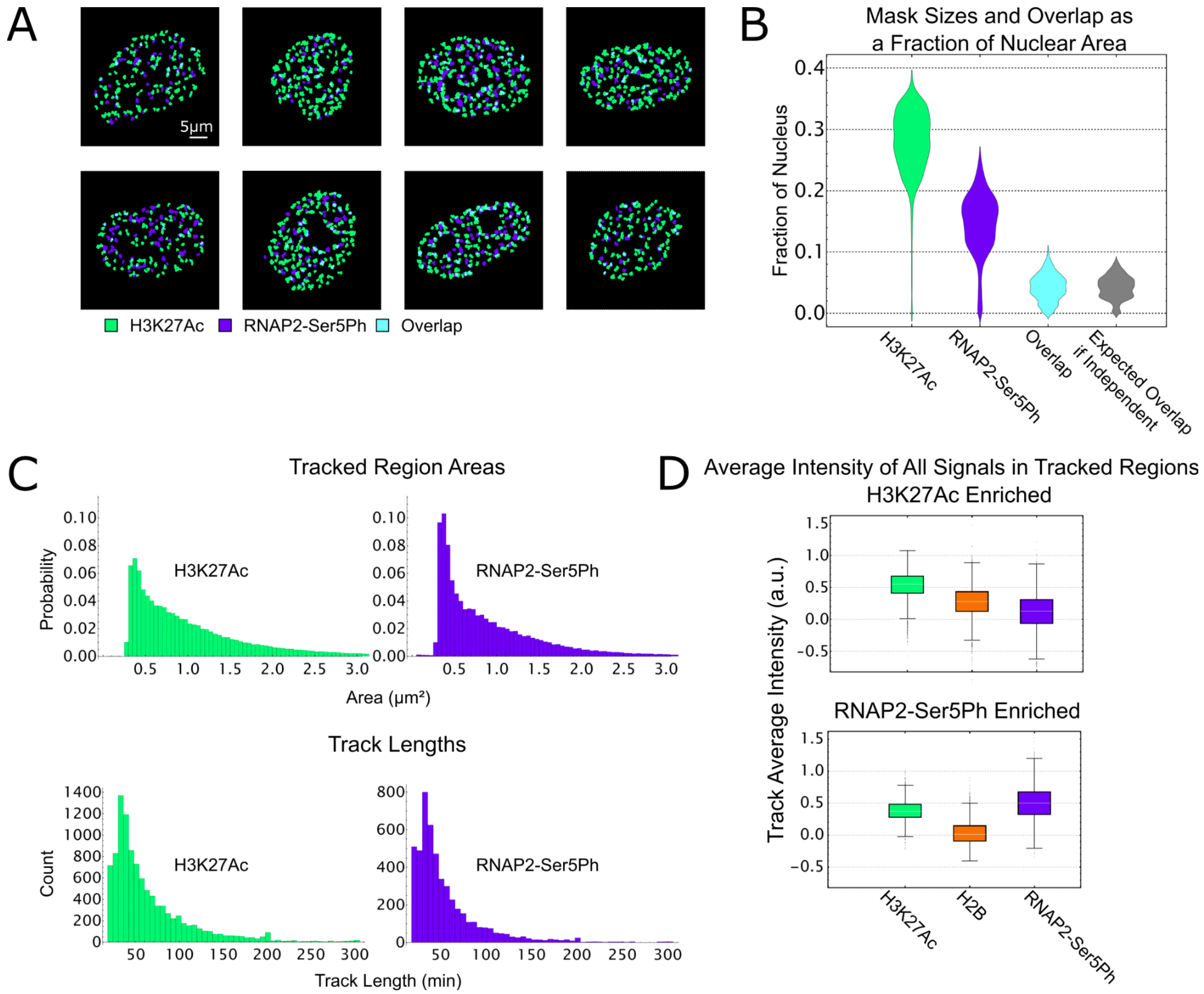

Fig. S3: Characterization of H3K27ac and RNAP2-Ser5ph Regions: Overlap, Nuclear Area, and Intensity

A. Sample images from 8 cells demonstrating the location and overlap between H3K27ac and RNAP2-Ser5ph masks.
B. Distribution of mask area as a fraction of nuclear area, with the overlap between masks also shown. Expected overlap if independent represents the amount of expected overlap if each mask's area were independent and uniformly distributed throughout the nucleus. n=27 cells, 2700 masks.
C. Top) Histogram of area of detected regions for both H3K27ac and RNAP2-Ser5ph masks. n=228,610 regions for H3K27ac, n=143,854 regions for RNAP2-Ser5ph. Bottom) Histogram of track lengths for both H3K27ac and RNAP2-Ser5ph regions.
D. Distribution of average intensities in all signals for all H3K27ac and RNAP2-Ser5ph tracks (i.e. each track's signal is averaged, and contributes one data point to the distribution).

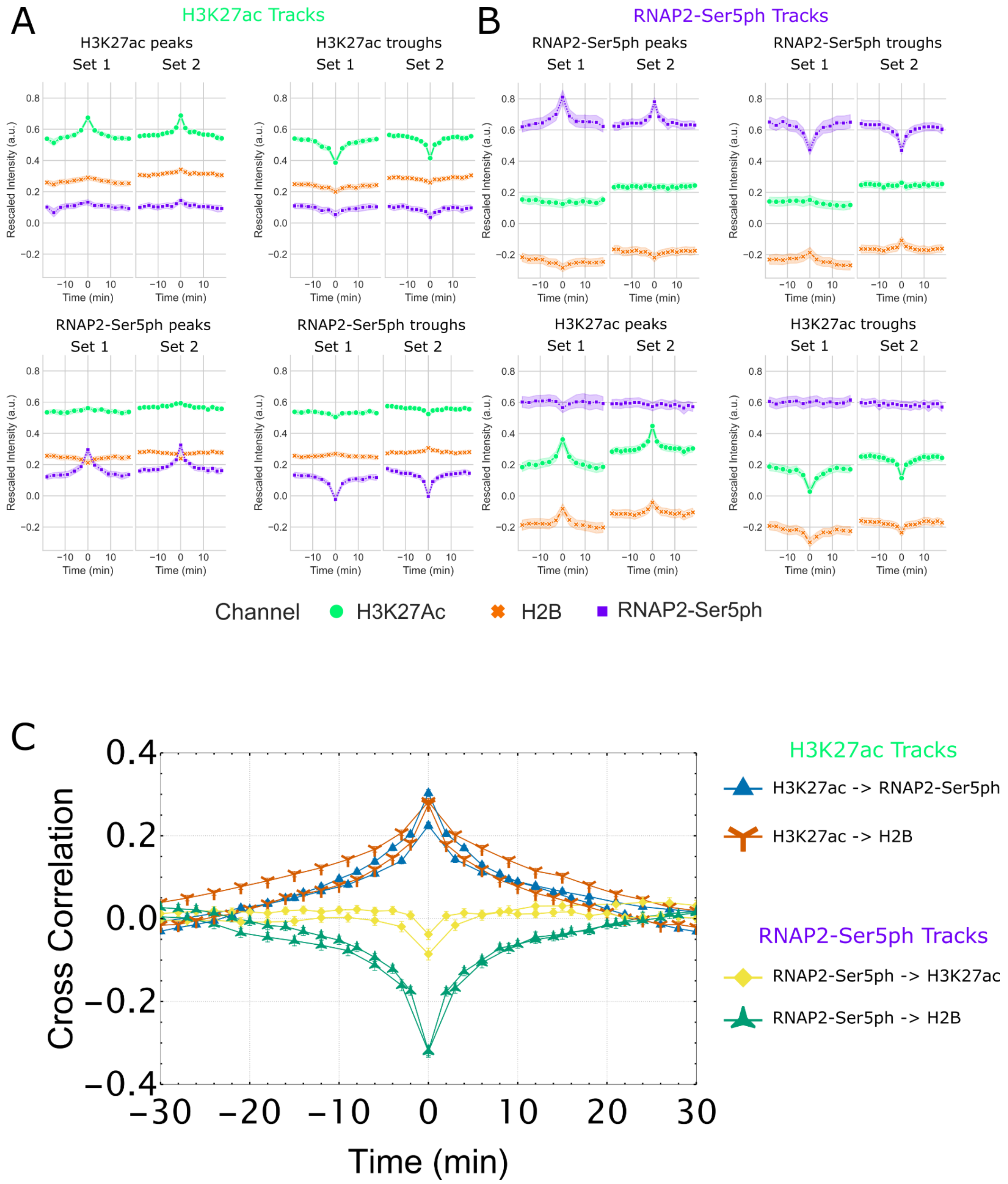

Fig. S4: Peak and Trough Detection Combined with Cross-Correlation Analysis Verify H3K27ac, RNAP2-Ser5ph and H2B Dynamic Behavior

A. Detected peaks and troughs in all H3K27ac enriched tracks for two data sets of n=13 cells and n=15 cells. Both RNAP2-Ser5ph and H3K27ac peaks and troughs were detected, and all 3 signals were aligned based upon those peaks and troughs.
B. Same as (A), for RNAP2-Ser5ph enriched regions.
C. Cross correlation of various signals inside of H3K27ac and RNAP2-Ser5ph enriched regions, calculated for all tracks of length 20 frames or greater. Error bars are standard deviation obtained via bootstrapping (n=1000).

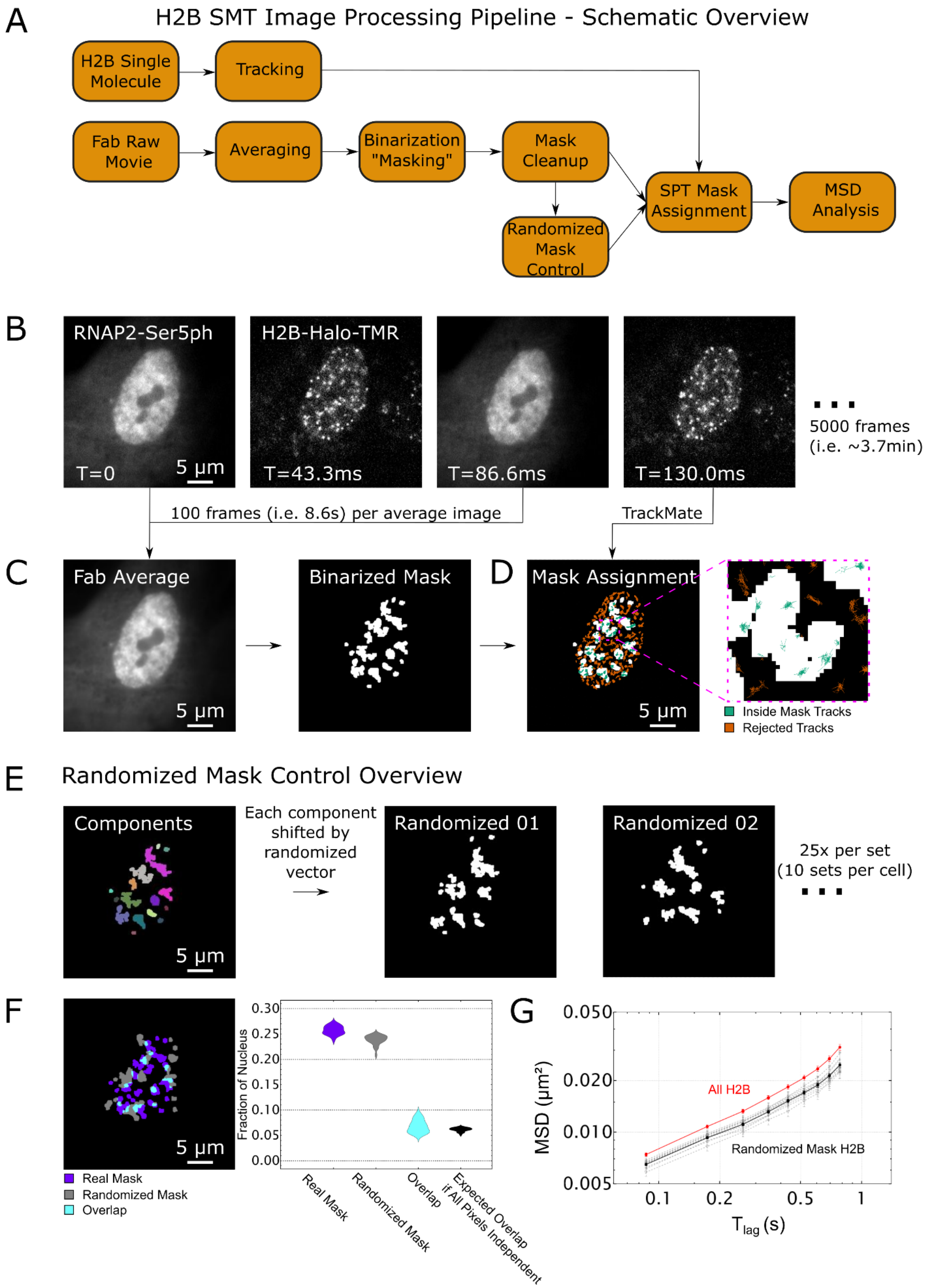

Fig. S5: Analysis Pipeline for Tracking Single Nucleosomes in the Context of Post-Translational Modifications

A. Schematic overview of the image processing pipeline for H2B single-molecule tracking experiments

B. Fab and single-molecule H2B are imaged in an alternating fashion, for 5000 total frames (3.7 min).
C. 100 Fab images are used to create an average intensity image, reducing the noise generated by freely diffusing Fab. A local adaptive binarization algorithm is then applied to create a mask of enriched regions.
D. Determination is made whether single nucleosome tracks are inside or outside of the mask, and tracks outside of a mask are not used in subsequent analysis.
E. To create a randomized mask, first the connected pixels in a mask are segmented into separate regions, or “components”. Entire components are shifted by an individually randomized vector, creating a mask with random distribution of components, but whose morphology is analogous to the original mask.
F. Overlap between real and randomized masks. Distribution of overlap is quantified and compared to an *in-silico* control, the expected overlap between masks if all pixels in the nucleus were randomized individually. n=25 real and randomized masks
G. Log-log plot of all nuclear H2B MSD in a single cell vs those inside of 10 sets of 25 randomized masks for the same cell. The EA-TA-MSD for H2B inside randomized masks is always slightly decreased, demonstrating the need for a randomized mask control.

Supplementary Movies

Movie S1. Simultaneous live-cell imaging of H2B, RNAP2-Ser5ph, and H3K27ac in living cells for up to 300min. To accompany figures 1 and 2.

Movie S2. Masking of RNAP2-Ser5ph enriched regions combined with single-molecule tracking of nucleosomes in living cells. To accompany figure 3.

Movie S3. Masking of H3K27ac enriched regions combined with single-molecule tracking of nucleosomes in living cells. To accompany figure 4.
